# Classification of body postures using smart workwear

**DOI:** 10.1186/s12891-022-05821-9

**Published:** 2022-10-18

**Authors:** Christian Lins, Andreas Hein

**Affiliations:** 1 Department of Computer Science, Hamburg University of Applied Sciences, Hamburg, Germany; 2 Department of Health Services Research, Carl von Ossietzky University Oldenburg, Oldenburg, Germany

**Keywords:** Non-neutral postures, Work-related musculoskeletal disorders, Inertial sensors, Neuroevolution

## Abstract

**Background:**

Despite advancing automation, employees in many industrial and service occupations still have to perform physically intensive work that may have negative effects on the health of the musculoskeletal system. For targeted preventive measures, precise knowledge of the work postures and movements performed is necessary.

**Methods:**

Prototype smart work clothes equipped with 15 inertial sensors were used to record reference body postures of 20 subjects. These reference postures were used to create a software-based posture classifier according to the Ovako Working Posture Analysing System (OWAS) by means of an evolutionary training algorithm.

**Results:**

A total of 111,275 posture shots were recorded and used for training the classifier. The results show that smart workwear, with the help of evolutionary trained software classifiers, is in principle capable of detecting harmful postures of its wearer. The detection rate of the evolutionary trained classifier ($\bar{a}_{ccr} = 0.35$ for the postures of the back, $\bar{a}_{ccr} = 0.64$ for the arms, and $\bar{a}_{ccr} = 0.25$ for the legs) outperforms that of a TensorFlow trained classifying neural network.

**Conclusions:**

In principle, smart workwear – as prototypically shown in this paper – can be a helpful tool for assessing an individual’s risk for work-related musculoskeletal disorders. Numerous potential sources of error have been identified that can affect the detection accuracy of software classifiers required for this purpose.

**Supplementary Information:**

The online version contains supplementary material available at 10.1186/s12891-022-05821-9.

## Introduction

**Fig. 1:**
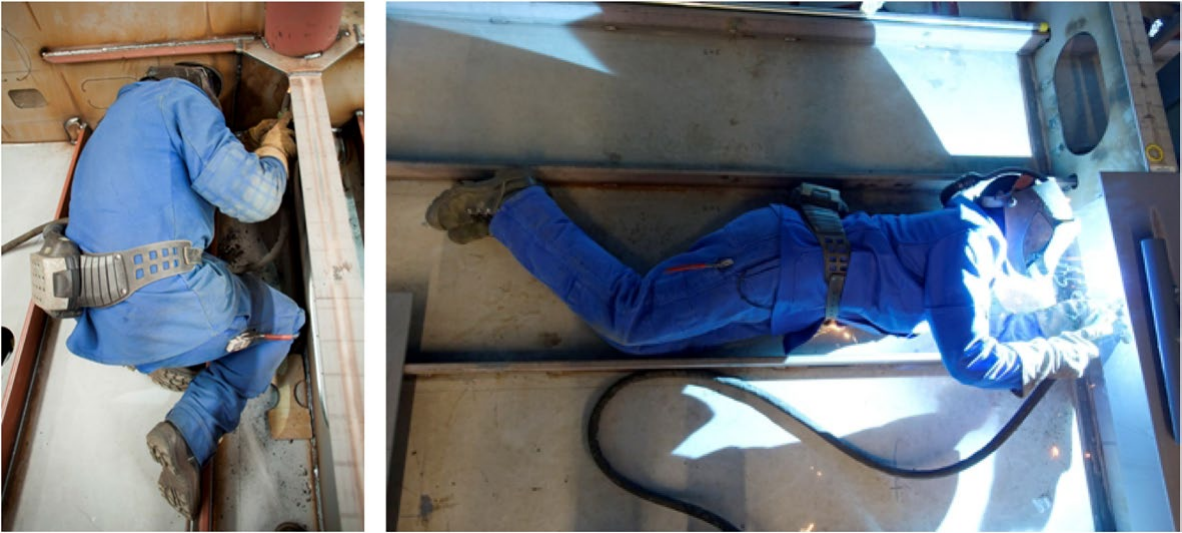
Shipyard welders in non-neutral positions (Image source: used by courtesy of MEYER WERFT GmbH & Co. KG.)

Despite the progress of automation in the economy, many employees still have to perform physically intensive and potentially dangerous tasks in their daily work. Striking examples are the metal and heavy industries (see Fig. 1), but also service sectors such as logistics, emergency services [1], or nursing care [2].

These physically intensive activities often involve postures that can have a negative impact on the musculoskeletal system. If such postures and movements are performed regularly or over a long period of time, they are a risk factor for musculoskeletal disorders (MSD). MSD are often the result of heavy physical work, such as manual handling and (heavy) lifting, or of physical misconduct (such as non-neutral postures) that is repeated over long periods of time [3–5]. The most common MSD include, for example, chronic back pain or knee joint atrophy [6–8].

Work-related MSD are a major cause of absenteeism and early retirement among workers [9]. The total annual cost of lost productivity due to work-related MSD is estimated to be 2% of the GDP in Europe alone [10]. Treatment of MSD imposes significant costs on public health systems in various countries, e.g., Germany’s Federal Statistical Office reports a cost of 420 € per citizen per year (year 2015) [11, 12].

The risk of MSD can be reduced by appropriate preventive measures and the effects of MSD can be reduced by countermeasures. This requires a.) detailed knowledge of the workplace in general, b.) the individual’s health status, and c.) the specific postures and movements. Thus preventive measures are a necessity, e.g., in the context of occupational health management in industrial companies with physically hard-working employees, in particular to minimize the costs of production losses due to workforce illnesses or early retirements. In order to take targeted and individualized preventive measures or tailored, personalized interventions such as redesigning workplace structures and shift schedules, it is essential to obtain an accurate overview of the nature and frequency of the non-neutral postures [13].

Today, checklists and more specific assessment methods, among others, are used to assess individual risks and should enable the person observing to assess activities and workplaces. The findings of these assessment methods can be used to derive appropriate, workplace- and person-specific prevention measures in the context of situational and behavioral prevention [13]. One of these assessment methods is the Ovako Working posture Analysing System (OWAS) [14], which is later described in detail.

In addition to optimizing the (individual) work environments of employees in the context of health prevention, targeted physical training is also an important aspect in the prevention of MSD. Trained employees are less prone to physical misconduct or are better able to compensate for it, so that they also have a lower risk of MSD.^1^

Manually recording the postures in the work environment is time-consuming and error-prone. A technology-supported measurement system could therefore help to improve the situation. In addition to the validity of the measurement, a particular challenge is the special requirements placed on the measurement system in everyday working life. Ideally, even in restricted spaces without human observers, it should provide information about the relevant postures unobtrusively but continuously.

A measuring system integrated into the workwear can meet these requirements. We will present the prototype of such a measuring system (named SIRKA [18]) in this paper. For the data analysis, the integration of the sensors in the clothing and not on the body is a particular challenge, since the drape of the clothing and the distance to the body of the wearer induce additional errors in the measurement. For this reason, the approach presented here uses an evolutionary training algorithm that is characterized by high adaptibility and flexibility to create a classifying neural network.

Body-worn motion capture systems like the one presented here are particularly useful here, as they do not suffer from occlusion of individual body parts. Nevertheless, body-worn motion capture (MoCap) systems must not interfere with work or disturb everyday life, as this would reduce acceptance on the one hand and falsify the measurement as such on the other. MoCap systems that can be integrated into clothing are therefore suitable for long-term measurement that is as inconspicuous as possible. The SIRKA system (see Section 3.1) was therefore chosen for implementation in this practical application.

In the long term, our approach can be used to obtain a realistic and comprehensive picture of body postures and movements in the long term in everyday work.

To summarize the contributions of this paper:

1. An approach to automatically and continuously assess the MSD risk based on a posture classifying software model that uses motion capture data. Qualitative observations using the OWAS method by human raters are mapped into quantifiable ratings using this model.
2. We show how data from a motion capture suit integrated into work clothing can be used to derive a skeleton model from it.
3. An evolutionary approach to train an artificial neural network (ANN) to classify posture using an evolutionary algorithm that learns from human observations and can deal with uncertainties in data acquisition (e.g., no fixed joint angles).

The following paper is structured as follows: in the following Section 2 related approaches to assess the risk for MSD both manual (see Section 2.1) and technology-driven (Section 2.3) are reviewed and the OWAS method, which is the basis for our classifier, is presented in detail (Section 2.2). In Section 3 the general approach, the motion capture system, the data analysis, and details of the evaluation study are described. Section 4 contains the results of the evaluation and is followed by the discussion in Section 5. In Section 6 the paper is summarized and concluded.

## Related Work

First, manual assessment methods, i.e., those that can usually be done with pen and paper, are reviewed for assessing postures in the context of MSD (Section 2.1). In doing so, we motivate the choice of the OWAS method for our approach and present it in more detail (Section 2.2). Finally, we present some work that already targets technology-assisted assessment of postures (Section 2.3).

### Manual approaches to assess Musculoskeletal Disorders

Numerous paper-based assessment methods exist for a wide variety of applications. Some are intended for individual parts of the body or for specific medical conditions. Here, only general methods that consider postures of the entire body and determine overall risk for MSD will be considered further. Takala et al. [19] provide a systematic review of observational methods for assessing biomechanical exposure at work (see Table 1).

Only a few assessment methods in Table 1 were reported to be associated with musculoskeletal disorders. The association with MSD is critical for assessing the validity of the method, that is, whether the assessment method actually measures the risk for MSD.

The second aspect is reliability, i.e., the test-retest reliability of the measurement when repeated. A distinction is made between *intra*-observer reliability (one observer assesses at multiple time points) and *inter*-observer reliability (multiple observers assess at a given time point). Thus, selection criteria for using an *inter*-observer method should be association with MSD and reliability.

One such established and often pen-and-paper assessment method to which these criteria apply is the OWAS method [14, 20]. The OWAS method, as originally described by Karhu et al. [14], seems widely used^2^ and is applied to assess physical risk factors for musculoskeletal disorders. The validity of risk assessment using OWAS with respect to MSD has already been demonstrated for back pain: Burdorf et al. [22] confirmed a weak correlation between OWAS assessments and subsequent prevalence for back pain as a common variant of MSD (approximately 40% of MSD complaints [8]). The procedure within the assessment of postures using the OWAS method is described in more detail in the following section.

**Table 1:** Overview of paper-based assessment method for postures (whole body), expanded from Takala et al. [19]

Method, Year of publication	Metrics	Observation strategy	Associated with MSD	Intra-/Inter-observer Reliability
Ovako Working Posture Assessment System (OWAS), 1977 [14]	Frequency of postures	Sampling over time	Yes [22]	Good/Good
Arbeitswissenschaftliches Erhebungsverfahren zur Tätigkeitsanalyse [Occupational science survey method for activity analysis], 1979 [23]	Characterization of the postures	-	-	-/-
Posture targetting, 1979 [24]	Frequency of postures	-	-	-/-
Plan för identifiering av belastningsfaktorer (PLIBEL) [Method for the identification of ergonomic stress factors], 1995 [25]	Yes-/No-Questions; Characterization of the postures	Selection according to knowledge about the activity and observations	-	-/Moderate
Posture, activity, tools and handling (PATH), 1996 [26]	Time in certain postures	Sampling over time	-	Moderate-Good/Moderate-Good
Quick exposure check (QEC), 1999 [27]	Total weighted posture score	“Worst case” of posture	Yes	Moderate/Moderate
Rapid entire body assessment (REBA), 2000 [28]	Total weighted posture score	Typical postures, with load or over a long period of time	-	-/Low-Moderate
Washington State ergonomic checklists, 2000 [29]	Yes-/No-Questions about typical work	Screening by task classes	Yes	-/Moderat
Chung’s postural workload evaluation, 2002 [30]	Evaluation of postural complaints	-	-	-/-
The European Assembly Worksheet (EAWS), 2012 [31]	Total score from (repetitive) work, postures, and forces	Evaluation of typical activities	-	-/-

### OWAS method

The *Ovako Working Posture Analysing System* (OWAS) was developed in the 1970s after systematic observation of workers in a Finnish steel mill (*Ovako Oy*). The aim was to develop an easy-to-use and practical assessment method for risky work postures [14]. Based on photographs of steelworkers’ work postures, 84 typical postures were identified and assigned a risk class representing the risk for musculoskeletal disorders [32].

**Fig. 2:**
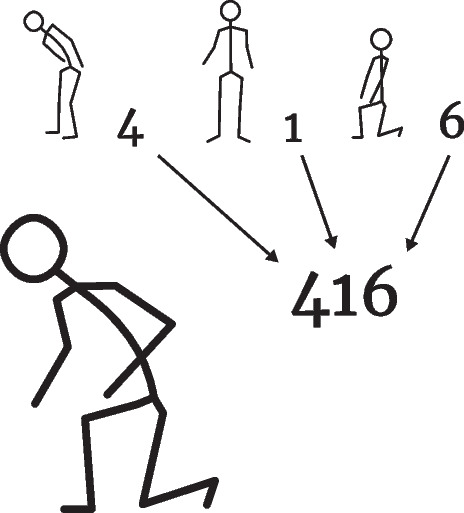
An example OWAS posture, where the three-digit OWAS code is composed of the ratings of the partial postures

As part of an OWAS observation, postures are classified into three categories: Back, Arms, and Legs. Each category includes the postures listed in the Table 2–4. In each of the three categories, the observing person selects the partial posture that most closely matches the subject’s actual posture. Each partial posture is assigned a corresponding numerical code. The codes of the back, arms, and legs (in that order) together form a three-digit code that describes the posture (see Fig. 2 for an example). There are 72 possible combinations for this OWAS code (if you exclude leg posture 7, since it is actually a movement). There are OWAS extensions with a fourth digit for the applied force or the load [14, 32].

In OWAS, subjects’ postures are assessed at a fixed time interval (usually between 30 s – 5 min) by an observing person [33].

**Table 2:** OWAS category *Back*

Category	Code	Posture	Description
Back	1xx		Back straight
	2xx		Back bent
	3xx		Back twisted
	4xx		Back bent and twisted

**Table 3:** OWAS category *Arms*

Category	Code	Posture	Description
Arms	x1x		Arms below shoulders
	x2x		One arm at or above shoulder height
	x3x		Both arms at or above shoulder height

**Table 4:** OWAS category *Legs*

Category	Code	Posture	Description
Legs	xx1		Sitting
	xx2		Standing/load on both straight legs
	xx3		Standing/Load on one straight leg
	xx4		Standing/Load on bent legs
	xx5		Standing/Load on one bent leg
	xx6		Kneeling on one or both legs
	xx7		Walking

Within the OWAS method, each posture is assigned an action class (from 1 to 4) indicating the urgency of response to the detected posture, i.e., to what extent countermeasures should be initiated. Action class 1 indicates the least or no risk, while action class 4 is used for postures that require immediate countermeasures. Table 5 shows the associated risk classes as a function of the three limb categories and the optional load/force component.

**Table 5:**
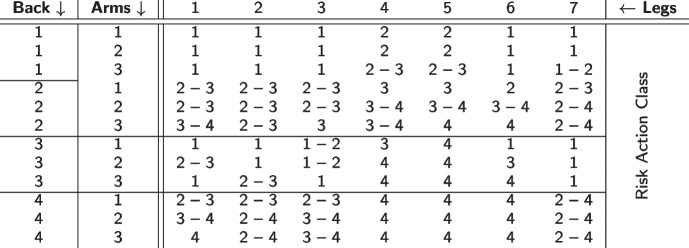
OWAS Risk Action Classes (as of [32]). The numbers of the *back* and *arms* columns and the *legs* row refer to their respective OWAS codes. The risk action class depends on the application of force, hence the intervals for some combinations

The OWAS method is a comparatively simple assessment method and thus predestined for use by personnel not explicitly trained in ergonomics. Weir et al. [34] were able to show that for video-based observations the classification accuracy of people with and without ergonomics training does not differ significantly. This has also been shown for live human observations, i.e., not video recordings [13].

### Motion capture-based classification of body postures

Various approaches exist for the analysis of body postures with motion capture systems or other technical support. Some selected approaches are briefly described below, with a focus on the OWAS method.

There are several approaches to using OWAS using technical systems: Mattila and Vilkki’s early solution [32] falls into the category *computer-assisted*. It consists of two terminal programs; OWASCO for data entry and OWASAN for analysis of the entered data. The input is done by the person observing with the help of the software. The software reminds the moment of observation with an acoustic signal.

Gudehus [35, 36] uses OWAS in combination with a software-based ergonomic evaluation system. Here, the classification of postures based on motion capture data is rule-based with threshold/cut-off values of selected angles. Thus, no human evaluation is included, but the angle, e.g. for a bent back, is arbitrarily predefined.

Diego-Mas and Alcaide-Marzal demonstrate a way to use the Kinect sensor to evaluate postures with OWAS [37]. Their system uses fixed angles at each joint to then make rule-based decisions about OWAS posture. The authors then compare the classification results to the ratings of human observers who classified the postures based on photographs. The authors highlight that the accuracy of the Kinect’s skeletal tracking increases greatly as the angle between the sensor and the sagittal plane of the subject increases.

Li and Xu [38] use an artificial neural network to classify based on skeletal data projected onto a 2D plane using the RULA method. The structure of the network is fixed. Difficulties stated were that due to lack of sufficient training data with an unbalanced^3^ dataset had to be worked with.

Nath et al. [39] present a simple approach to observe work postures. They use two Android smartphones to classify typical work postures using the smartphones’ accelerometers. Mathematically, the orientations of the smartphones are determined using the gravity vector of the accelerometer. Only the bending of the back and the angles at the arms and elbows are considered. The risk assessment of the posture is then made on the basis of the calculated angles.

To the best of the authors’ knowledge, there is no known approach to date that uses data recorded by smart workwear to perform an assessment of postures using an evolutionary learned classification model.

## Methods

**Fig. 3:**
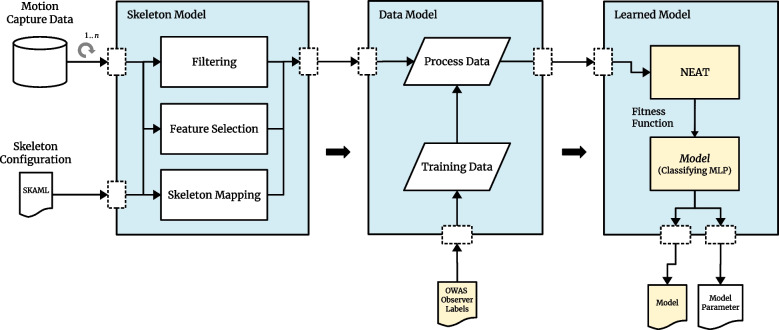
Overview about the approach to create the classification models

The process illustrated on Fig. 3 is intended to create and optimize the smallest possible classifying multilayer perceptron network so that it can process motion data from a simplified human skeleton based on orientations provided by the SIRKA system. As a result, the network should then classify and output the corresponding body posture according to the OWAS method. Here, the motion capture data is provided by the SIRKA system (Section 3.1), and the labels come from the study with human participants and observers described in Section 3.2. The result of the process is a classifying MLP network.

The individual processing steps of the process are described in detail in Section 3.3, followed by some remarks concerning the model training methodology (Section 3.4).

### Inertial measuring suit SIRKA

**Fig. 4:**
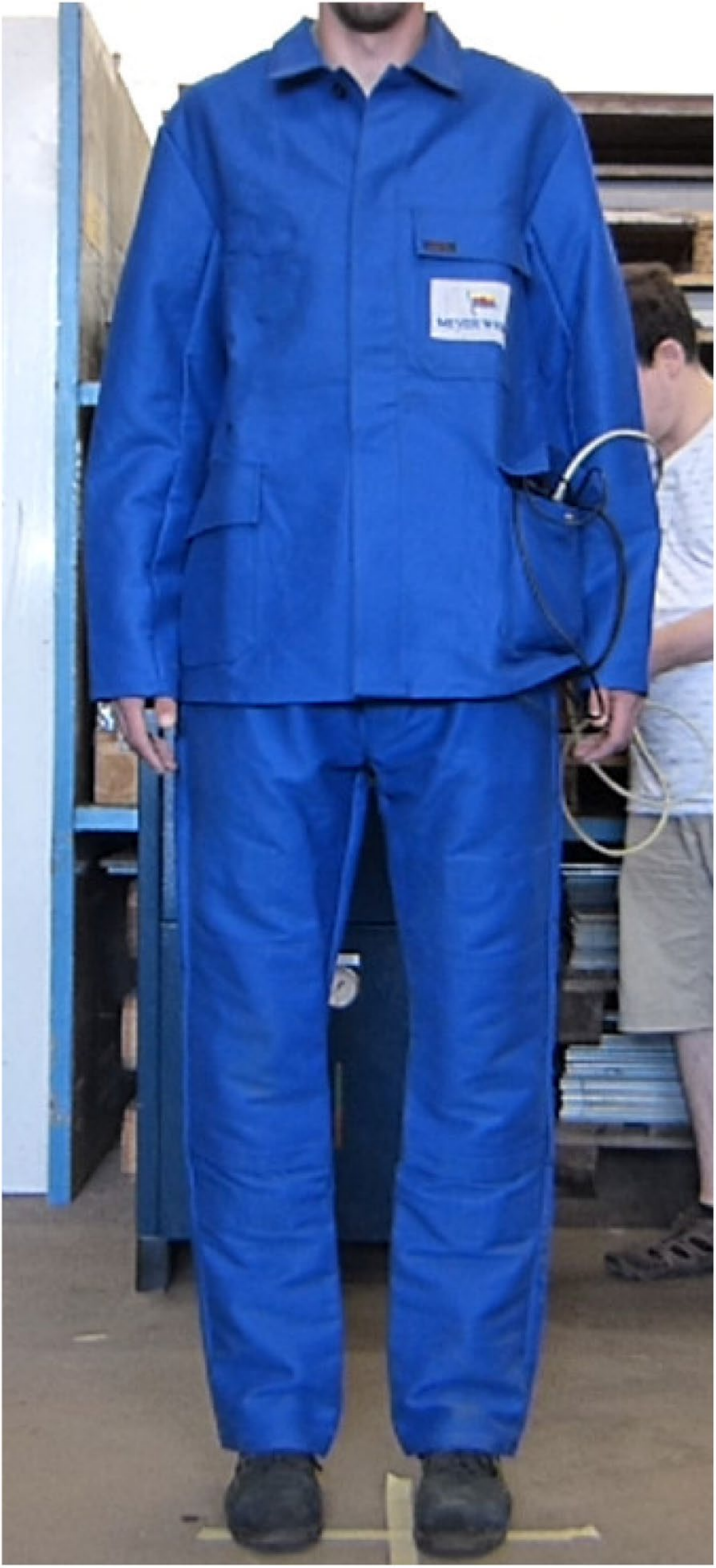
SIRKA measuring suit

The SIRKA suit (see Fig. 4) was developed in the research project of the same name and is the prototype of an inertial measurement suit that resembles smart workwear. The suit has two distinct features, which were also development goals of the project: first, the sensor nodes of the suit are encapsulated waterproof and can be integrated into work clothing, and second, the sensor fusion does not require a magnetometer, which makes the suit insensitive to magnetic interference (e.g., large amounts of steel as in shipyards) [18, 40, 41].

**Fig. 5:**
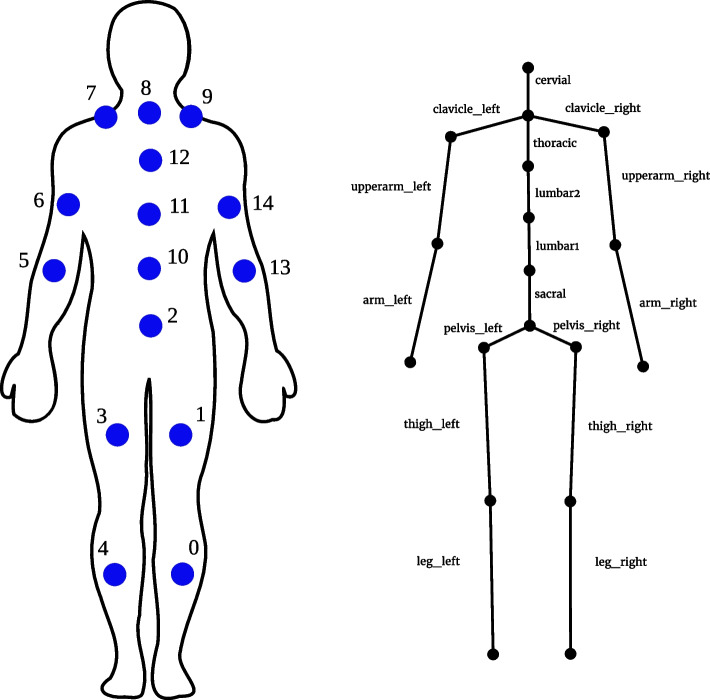
SIRKA sensors and skeleton. **a** Placement of the sensor nodes in the SIRKA suit (according to [41]). The numbers represent the internal IDs of the nodes in the SIRKA system. **b** SIRKA skeleton with bone labels

The SIRKA system consists of a total of 15 sensor nodes (each containing an ARM Cortex M3 core together with an Bosch BMI160 Sensortec 6-axis inertial sensor unit) divided into two logical strands – one each for upper body and lower limbs (see Fig. 5). The gyroscope of the IMU is configured with $\pm 1000^{\circ }/s$ and the acceleromter with $\pm 8g$ with both 16-bit fixed-point values [41]. The sensorboards are electrically coupled via a cable harness and connected to a central embedded system via several RS485 bus systems, where the sensor fusion^4^ combines the data from the individual sensor nodes (see Fig. 5). The maximum orientation error of the sensor fusion was determined with $< 5^{\circ }$ [41, p. 107].

Unlike most other inertial measurement systems, in this system here the sensors are integrated together with their cables for data connection and power supply on the inside of the work clothing. For this purpose, suitable inner pockets that can be secured with hook-and-loop fasteners have been incorporated into the clothing.

When designing the SIRKA system, particular attention was paid to its practical suitability in everyday industrial work. The cabling, the sensor nodes, and the central unit with sensor fusion are integrated into the work clothing in such a way that they are (virtually) unnoticeable when worn and do not interfere in any way. In contrast to other IMU-based MoCap systems such as the commercial Xsens MVN, limitations in precision were thus accepted in order to increase the system’s practicality. In many cases, the sensor nodes are not located on the limbs whose orientation is to be measured, but at a distance in the clothing above them. Work clothes are usually cut wide with stiff fabric, so that additional errors are induced in the measurement by draping and unintentional movement of the cloth.

The orientation estimates of the sensor nodes are saved to the computer’s memory card and can be taken from there after recording and processed further.

### Study setup

In the study 20 participants were separately observed by three varying human raters while posing in each of the 72 (excluding lower limbs posture 7 ‘walking’) OWAS postures (see Tables 2, 3, 4).

The 20 participants in the study each wore a SIRKA suit that was individually calibrated to each person. During the trial, the suit recorded the subjects’ movement patterns on the SIRKA central processing unit’s memory card.

The three raters were equipped with pens and structured sheets and viewed each participant from one viewing-angle. The raters sat next to each other at a distance of around 1m among each other, and were requested to remain seated, to ensure the independent evaluation of the raters [13]. The constellation of the rater-group was altered per participant.

The participants wore authentic industrial workers’ workwear, typically made of robust and wide cut fabric, which realistically complicates the recognition of postures. The participant was asked to model the postures independently and to align himself or herself frontally with the raters. The study director explicitly did not correct the postures in order not to influence the raters.

Since neither participants nor raters have been previously trained in OWAS, both participants and raters received a short introduction in the OWAS method.

*Process of one trial*

1. A custom software generates a random OWAS code using a Pseudo-RNG initialized with the participant number. Within one trial the generated OWAS code can only occur once. The OWAS posture is displayed to the participant modeling the posture, but not to the raters.
2. The participant interprets the posture and performs it independently. The raters can observe the participant while he or she models the posture.
3. The three raters are given up to 30 s to rate the posture independently (no communication between the raters was allowed) using a paper sheet. They are not allowed to stand up, but they may move their upper body and adjust their view of the participant.
4. Steps 1–3 are repeated for every one of the 72 posture combinations.

### Data processing steps

#### Skeleton mapping: A kinematic tree from sensor data

The sensor nodes of the SIRKA system provide their raw data to a central embedded computer, which is usually placed in the jacket pocket of the suit. The sensor fusion software provides an estimate of the orientations of all sensor nodes or the corresponding body segments in the predefined SIRKA skeleton with a frequency of 10Hz (see Fig. 5 (b)).

For the SIRKA skeleton, only the orientations of the individual sensor nodes are available. Once the orientations of the sensors are assigned to the individual bones, a kinematic tree can be calculated starting from the origin (*root*). The origin of the SIRKA skeleton is an imagined central joint in the pelvic region (called *pelvis* in the skeleton definition). The absolute position in space is not known because the SIRKA suit, as an inertial measurement system, does not provide it. Usually, the position of the origin is set to $$\mathbf {p}_0 = (\begin{array}{ccc}0&0&0\end{array})^ T$$. Starting from this origin, the bones of the skeleton are traversed in latitudinal search, i.e. first the three bones connected with *pelvis* are considered. Their origin $\mathbf {p}_{i,start}$ is known with the position of *pelvis*, searched is the position $\mathbf {p}_{i,end}$ of the endpoint of the *i*th bone. This position depends on the direction vector $\mathbf {d}_i$ and the length $l_i$ of the bone and can therefore be determined as follows:

1 $$\begin{aligned} \mathbf {p}_{i,End} = \mathbf {p}_{i,Start} + \mathbf {d}_i \cdot l_i \end{aligned}$$

The procedure is completely described in Algorithm 1 in pseudocode. With this, the skeleton can now be moved based on the sensor data.

**Figure:**
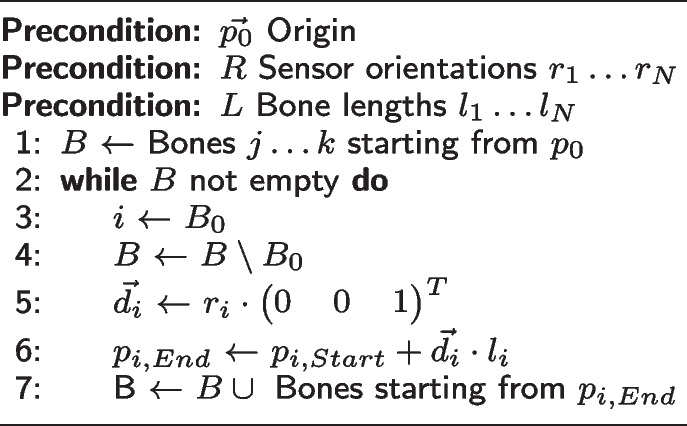
**Algorithm 1**Kinematic Tree

The algorithm is run for each new data frame.

#### Feature selection

For the data model and classification, a rotation and translation invariant representation of the skeleton’s posture is needed, i.e. a representation that is independent of the position and orientation of the entire skeleton in space. Additionally, the representation should be independent of the length of the limbs. These requirements are met if using the angles at the skeleton joints.

First, the angles at the joints of the skeleton are determined, i.e. the angles between two connected bones. Based on the bones of the SIRKA skeleton (see Fig. 5), this results in 14 angles (i.e. features) as representation of a pose (see Table 6). This also reduces the dimensionality of the features compared to the raw sensor data (15 orientations represented as quaternions would yield 60 input features).

**Table 6:** Angle between bones as input features. The position of the bones in the skeleton is shown in Fig. 5 (b)

Index	Bone A	Bone B
1	clavicle_left	upperarm_left
2	clavicle_right	upperarm_right
3	lumbar1	lumbar2
4	lumbar2	thoracic
5	pelvis_left	thigh_left
6	pelvis_right	thigh_right
7	sacral	lumbar1
8	thigh_left	leg_left
9	thigh_right	leg_right
10	thoracic	cervial
11	thoracic	clavicle_left
12	thoracic	clavicle_right
13	upperarm_left	arm_left
14	upperarm_right	arm_right

An orientation in space can be represented as a rotation about a certain axis of rotation. Let *o*, *u* be two connected bones with rotation matrices $Q_o, Q_u$ from $SO_3$^5^ in the global coordinate system. Then the rotation of $Q_u$ in the coordinate system of $Q_o$ can be written as:

2 $$\begin{aligned} \tilde{Q} = Q_o^{-1} \cdot Q_u = Q_o^T \cdot Q_u \end{aligned}$$

The solution of the following equation system 3 is then the rotation axis (eigenvector *v*) of this rotation ($\mathbf {I}$ is the unit matrix with eigenvalue 1). A solution exists only if a rotation actually exists, i.e. $\tilde{Q} \ne \mathbf {I}$.

3 $$\begin{aligned} (\tilde{Q} - \mathbf {I}) v = 0 \end{aligned}$$

The angle of rotation $\varphi$ is then (note: the trace *tr* of a matrix is the sum of the eigenvalues, i.e., the diagonal values [42, p. 664])^6^:

4 $$\begin{aligned} \varphi = \text {arccos } (\frac{1}{2} (tr( \tilde{Q}) - 1)) \end{aligned}$$

The motion capture data is now pre-processed, available in a skeleton-based representation with selected features, and can be transformed into a suitable data model.

#### Training data from the inertial sensor data

The SIRKA suit provides orientations for the individual limbs, which are used to build the kinematic tree of a skeleton. These orientations are used here, after preprocessing described above, as inputs to a multilayer perceptron net whose topology and weights are created and optimized using the NEAT algorithm [43] (see Appendix 1.1).

*Labeling the data* In the study, the training data were recorded together with reference classifications from human raters. For each recorded posture, OWAS classifications are available from three raters each, which are assumed to be ground truth for the classification model.

With three observers, one cannot assume complete agreement between observers (*inter-rater reliability* [13]). Each OWAS category (back, arms, legs) is considered separately, and for each possible partial posture, a column containing the majority decision of the raters was added. The experiment was conducted with $N=3$ observing human raters.

*Partitioning of the training data sets* Usually, the dataset used for training an ML algorithm is divided into three parts:

1. Training data: the largest part of the data set is used to perform the actual training. As a rule, an error function is minimized here.
2. Test data: To avoid overfitting of the ML model to the training data, the quality of the model is checked regularly (e.g. after each epoch) using the test data. Usually, the test data is much smaller in size than the training data (e.g. 10% of the total data set).
3. Evaluation data: the evaluation data is used to perform the final assessment of the ML model. This data has not yet been seen by the ML model during training, so it can be used to realistically assess how well the model has been abstracted from the training data to unknown data.

In the neuroevolutionary NEAT algorithm used here, a division into three parts is not necessary. The training and test data can be combined here because the adjustment of the connection weights in NEAT is not directly coupled to values of the training data, but is done meta-heuristically. The overall fitness of the network is considered; it is not so straightforward to adjust a single weight based on a training dataset (as it is possible, for example, in the backpropagation method for training ANN). In this work, therefore, the dataset is first split into two parts: 90% test data and 10% evaluation data.

*Balancing the training data* Training data for machine learning must contain each target category in the same relative frequency as the target category occurs in reality, i.e., they must follow the same distribution. For the individual OWAS categories, one assumes that each posture occurs with equal frequency. Otherwise, the ML algorithm would also learn the (wrong) frequency distribution and not let its decision be based only on the input values. In addition, if the training dataset is based on data from multiple subjects, it must also be balanced with respect to individuals, i.e., data from each individual must be equally distributed for each category.

For balancing the training data for the OWAS classifier, the method given in Appendix 1.2 was used. The algorithm is tolerant of subject combinations that do not allow for perfect balancing because, for example, too little data is available for specific categories. Additionally, the algorithm is heuristic when subsampling the data.

#### Classification model

The OWAS code describing a particular posture consists of three independent digits (categories back, arms, legs; see Section 2.2). One classifier could be created for the three-digit total code, but the complexity is reduced if a separate classifier is trained for each category^7^.

**Fig. 6:**
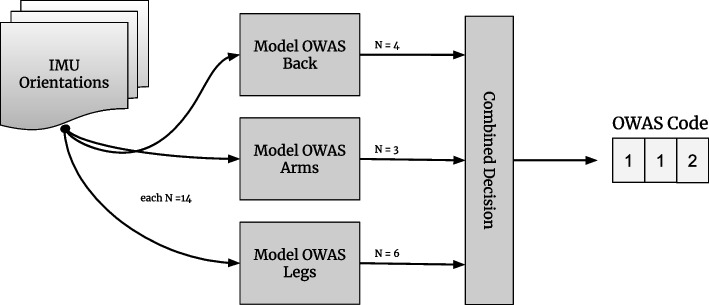
Structure of the OWAS classifier. The specification $N=14$ refers to the input features (skeleton angles) for the classifiers, the specifications to the right of the models refer to the number of output features

Figure 6 shows the structure of the classifier. The data from the IMU sensors are fed into three separately trained classification models, each of the models returns probabilities for all the partial postures of the category. The most likely partial (majority vote) postures can then be combined into an overall posture.

*Structure of the nets* The structure, i.e. topology of the ANN is learned in this use case with the NEAT algorithm. Nevertheless, there are constraints for the algorithm in which it searches for solutions. In particular, the input and output neurons are given by the problem.

Initially, all networks in a NEAT population do not have any hidden layers or neurons, but consist only of the given input and output neurons. At the beginning, all neurons are usually not connected either, only during the course of the algorithm the nets are mutated so that they also form connections between neurons.

The mutation operators of the NEAT algorithm randomly add new neurons and connections between neurons in the course.

*Error function* Evolutionary optimization requires an objective function. When training the ANN, the error is usually minimized, see Eq. 5:

5 $$\begin{aligned} \min 1 - \frac{N_{correct}}{N_{overall}} \end{aligned}$$

### Methodology of NEAT training

The evaluation run with the NEAT algorithm was performed on the High Performance Cluster (HPC) *CARL*^8^ of the Carl von Ossietzky University Oldenburg. The jobs were started with ten runs, since due to the reinitialization of the pseudo-random number generators the results differ in meta-heuristic procedures (Monte Carlo simulation).

The *Rectified Linear Unit* (ReLU) was used as the activation functions in both cases. The target function described in Eq. 5 was used.

Each job was run with a time limit of 2 days, 1 HPC node with 24 threads each, and 32GB RAM was started. The software used was YAHNI/0.9^9^ on Java/11.0.2.

## Results

To show the validity of the human observers’ assessments, inter-rater reliability was determined using Fleiss’ kappa for each OWAS category. The kappa values (a value of 1.0 indicates a perfect match) were as follows:

$$\kappa _{back} = 0.85, \kappa _{arms} = 0.98, \kappa _{legs} =0.85$$

The data of 6 participants were not usable (recording errors in the SIRKA system and miscalibrations of individual sensors), the data of the remaining 14 participants were – as described in the previous chapter – labelled with the observers’ labels and served as training and evaluation data for the NEAT algorithm.

At each start of the algorithm, data from 2 of the 14 participating individuals were randomly selected as evaluation data, and the remaining as training data. After each generation, the ANN with the highest fitness score was evaluated against the data from the two individuals unknown to the ANN.

**Table 7:** Confusion matrix for the comparison of the reference observations with the classification of the ANN for the postures of the **back**

Reference (human observers)					
ANN		1	2	3	4
	1 (Back straight)	**45% (95,808)**	10% (21,759)	33% (69,996)	11% (23,162)
	2 (Back bent)	8% (7,141)	**51% (47,095)**	12% (10,773)	29% (27,042)
	3 (Back twisted)	26% (23,630)	15% (13,482)	**44% (39,222)**	15% (13,231)
	4 (bent & twisted)	9% (19,431)	31% (66,231)	13% (27,791)	**46% (97,149)**

The boldface highlights the diagonal (true positive) values

**Table 8:** Confusion matrix for the comparison of the reference observations with the classifications of the ANN for the postures of the **arms**

Reference (human observers)				
ANN		1	2	3
	1 (Arms below shoulders)	**73% (165,843)**	19% (43,404)	8% (19,194)
	2 (One arm raised)	15% (31,235)	**64% (136,745)**	21% (44,908)
	3 (Both arms raised)	3% (3,836)	11% (16,304)	**87% (133,042)**

The boldface highlights the diagonal (true positive) values

**Table 9:** Confusion matrix for comparison of reference observations with ANN classifications for postures of **legs** (1: sitting, 2: standing with straight legs, 3: standing on one straight leg, 4: standing on both bent legs, 5: standing on one bent leg, 6: kneeling)

Reference (human observers)							
ANN		1	2	3	4	5	6
	1	**45% (79.856)**	10% (17.208)	8% (13.641)	17% (29.987)	9% (15.059)	12% (20.878)
	2	4% (4.832)	**38% (51.256)**	25% (34.213)	20% (26.735)	13% (17.937)	1% (1.681)
	3	9% (8.320)	22% (20.812)	**33% (31.363)**	17% (16.255)	16% (15.564)	2% (2.346)
	4	13% (15.438)	16% (18.377)	19% (22.255)	**35% (40.750)**	13% (14.716)	5% (6.008)
	5	7% (5.830)	16% (13.993)	20% (17.070)	12% (10.300)	**34% (29.683)**	11% (9.886)
	6	13% (34.240)	10% (27.485)	10% (26.872)	11% (30.171)	17% (45.000)	**40% (100.000)**

The boldface highlights the diagonal (true positive) values

The OWAS classifications of the trained ANN are now compared with the ratings of the observers. For this purpose, the classification results were compared in confusion matrices.

For this purpose, the best networks of each of the ten runs were tested against their respective training data. The summarized classifications are compared to the summarized ratings of the three observers. The human observations were combined in a majority decision. In the tables, relative values as well as absolute observations are given.

Table 7 presents the confusion matrix for the comparison of the reference observations (human observers) with the classifications of the ANN for the postures of the back. The diagonal values show that the ANNs agree with the reference in just under half (44–51%) of the posture ratings. The ANN have difficulty distinguishing the postures with straight and twisted backs (in 26% of the cases a straight back was recognized as twisted and in 33% of the cases a twisted back was recognized as straight). Analogous difficulties occur with bent backs and bent/twisted backs. In 31% of postures, a simply bent back is misclassified as bent/twisted. Conversely, in 29% of the cases, a bent/twisted back is classified as simply bent.

Table 8 presents the confusion matrix for the comparison of the reference observations (human observers) with the classifications of the ANN for the postures of the arms. The agreement between ANN and reference here is 64 – 87%. The ANN have difficulties in classification when an arm is raised or at shoulder height. In 19% of the cases the ANN does not recognize the posture (arms below shoulders) and in 21% both raised arms are classified as if only one arm was raised.

Table 9 presents the confusion matrix for the comparison of the reference observations (human observers) with the classifications of the ANN for the postures of the legs. In about one third of the cases, the classifications of the ANN and those of the human observers matched (see diagonal values of 33 – 45%). The following discrepancies are striking: in each of 13% of the cases a sitting posture was classified as “standing on bent legs” or “kneeling”. The posture “standing on straight legs” was misclassified by the ANNs as “standing on one straight leg” in 25%, as “standing on bent legs” in 20%, and as “standing on one bent leg” in still 13%. The posture “standing on one straight leg” was confused with the posture “standing with straight legs” in 25% of the cases. The posture “standing on both bent legs” was confused with the posture “standing with straight legs” in 20% of cases. The kneeling posture (OWAS code 6) was still confused by the ANN with the postures “standing on one bent leg” and “sitting” in 11% and 12%, respectively.

## Discussion

First, the results of the study are summarized and discussed. Then the possible causes of error for the non-optimal result are discussed and finally the results of the study are considered in the context of this work.

### General

The NEAT algorithm was used to test the generation of multilayer perceptron networks for the classification of OWAS body postures. Ten runs each were made up to generation 2000 for the postures of the back, arms, and legs. For each limb category, the classification accuracy (correct classification rate $a_{ccr}$) is different: after 2000 generations, the best ANNs (with evaluation data) achieve $\bar{a}_{ccr} = 0.35$ for the postures of the back, $\bar{a}_{ccr} = 0.64$ for the arms, and $\bar{a}_{ccr} = 0.25$ for the legs.

In general, the classification errors of the nets created with NEAT are too high for practical use. It remains to discuss the possible causes for the high classification errors in the following so that future approaches may avoid this obstacles.

### Error consideration

A number of possible causes of error come into question, which are now discussed starting from the representation of the reference posture by the human participants to the training of the classification networks.

#### Modelling the reference postures

In the evaluation study (see Sections 3.2), the participants were shown graphically and textually the postures they were supposed to adopt. The interpretation of the posture was entirely on the part of the participants, i.e., in case of a misinterpretation of the posture, the study director explicitly did not correct it in order to exclude any influence on the three independent observers. This is a possible cause for the deviation of the observations from the software reference. This cannot be a direct cause for the ANN error, because the label, i.e., marking which posture measured with the SIRKA suit belongs to which OWAS posture, was determined based on the observers and not on a reference.

Nevertheless, this circumstance may indirectly affect the quality of the training data: the reference software ensured that each posture was displayed only once. If the participant misinterpreted a posture, then one of the possible postures is missing from the data and another is duplicated. This imbalance in the target classes is corrected by balancing the training data (see Section 3.3.3), but in this process entries are removed from the data set if they disturb the balance. This reduces the amount of effective training data, which may well have a negative impact on the quality of the learned model.

#### Error of the measuring suit

Like any measuring system, the SIRKA suit also measures with a certain error. This measurement error is composed of the following components:

- Placement of the sensors in the suit: In the SIRKA system, the sensors are integrated in the work clothing and do not sit directly on the skin, as is often the case with comparable IMU systems. Depending on how close the clothing fits to the body, there is an offset of several centimeters between the clothing (i.e. sensor) and the moving limbs. This distance is not constant, but changes permanently during the movement. The distance is not uniform even when a movement is repeated, but can vary between participant and repetitions of similar movements due to random effects or minimal slippage of clothing. From a macro perspective, the posture looks the same to an observing person, but from the perspective of the raw sensor data, it can be completely different.
- Calibration error: Related to the previous point of sensor placement is the calibration of the system. Calibration involved determining the static offset of sensor placements on the human body for a given individual. This calibration is determined offline, i.e. in advance of the actual measurement. It was also not possible to correct the calibration during the measurement, as the SIRKA system does not currently provide for this. The calibration was performed days before the actual measurement, therefore the participants usually wore clothes of different thickness under the SIRKA work suit, which can lead to minimal calibration errors. More serious is when the calibration is not fully valid due to some other error and, for example, the orientation of a sensor was not correctly recorded. Non-valid calibrations usually lead to a defective recording.
- Noise error of the sensors: Due to minimal manufacturing tolerances in the production of the electronic components and due to different resistance behavior of the components at different ambient temperatures, minimal fluctuations of the sensor values occur during the analog-to-digital conversion. These fluctuations are noticeable as noise in the measured values.
- Error in the sensor fusion: SIRKA sensor fusion does not require data from a magnetometer. Thus, the system lacks a degree of freedom that can be compensated with the data of the logically connected accelerometers. If the participant stands still for a longer period of time (as in the case of an OWAS posture), there is hardly any acceleration data in the accelerometer beyond the noise (and gravity). This can lead to errors in orientation estimation [40].

#### Labeling the data

The human observers recorded the postures on structured paper observation sheets, which were manually digitized after the study. It cannot be ruled out that, despite great care, individual observations were not digitized correctly. This does not necessarily lead to errors in the classification of the ANN, but it may introduce contradictions in the data, for example, if a bent back is correctly marked as “bent” several times and incorrectly marked as “straight” once.

The SIRKA suit records the movements of its wearer as a continuous stream of motion capture data. In this data stream, the beginning and end of each posture was manually marked and assigned the corresponding data label. This manual process is subject to uncertainty because both the estimation *when* a posture begins and ends or when movement into a posture is complete is based on subjective human interpretation, as is the OWAS method, which does not specify angles or measurable properties. Therefore, it cannot be ruled out that minor inaccuracies or contradictions (cf. previous paragraph) may be introduced into the data due to misinterpretation.

#### Validity of the training data

It must be checked whether the data are valid, i.e. whether the data collected are fundamentally suitable for the problem to be solved. This can be estimated with the help of a probabilistic learning algorithm. In a decision tree, for example, the feature with the highest information density is used to decide which subtree to descend into. If a suitable algorithm (such as CARTs or ID4.5) is used to generate decision trees on the SIRKA data used here, one would expect plausible features to have the highest information density. For example, plausible features for deciding on a particular OWAS posture of the arms would be angles on arms or shoulders.

**Fig. 7:**
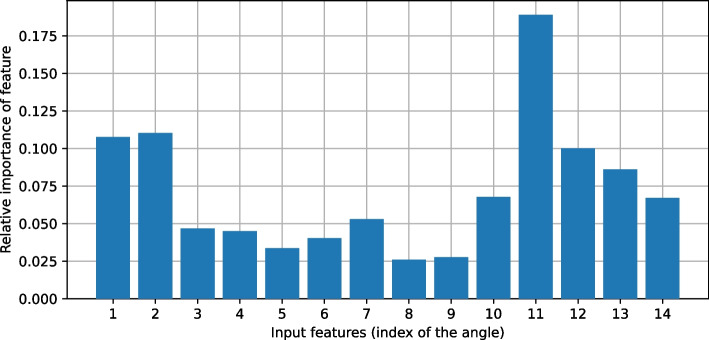
Relative importance of the individual angle features. Angle 11 is between left clavicle and upper back

This was tested as an example for the postures of the arms using the DecisionTreeRegressor implemented in Python from the scikit-learn package^10^. Figure 7 shows the relative importance of each body angle for the postures of the arms. To generate the graph, 12 of 14 participants’ data sets were randomly selected and the importance of the features was averaged over 100 runs.

The algorithms for generating the decision trees consider angle 11 (between left clavicle and upper back) to be the most significant for assessing posture, followed by angles 1 and 2, which are found between clavicles and upper arm bones on the left and right, respectively. This is quite plausible, since the bones mentioned are all located in the region of the upper body. The fact that the collarbones play a greater role here than the arms is probably due to the drape of the stiff fabric of the SIRKA suit, which significantly alters the position of SIRKA sensor nodes 7 and 9, especially when the arms are raised and lowered.

#### Balancing the training data

In the study discussed here – as is common practice – a portion of the data set was reserved as evaluation data with which the trained ANN was then compared. The selection of 2 out of 14 data sets was done randomly on each run of the algorithm. For an optimal classifying model, such random selection would be insignificant. Here, the scatter of results can be explained in part by this random selection.

In the training data, the distribution of classes should also correspond to the desired distribution of classes in reality. In the case of the OWAS postures, each posture should be equally likely to occur. To ensure that the randomly selected data of the participating individuals correspond to the equally distributed classes, balancing of the training data was performed (see section 3.3.3). The balancing algorithm involves a partial heuristic selection of data samples from the pool of available data and balances the classes with a certain tolerance. The tolerance is necessary because otherwise there would be too few data samples available. It is possible that the classification of rarely occurring classes is more often not correct if the training data is not balanced. Since the deviations here amount to a few percent, this error is considered rather small.

#### Learning algorithm

The size of the training data affects the ability of the algorithm to abstract general rules from the data. The training size of 12 data sets of the participants may be too small, but this also depends on the learning algorithm and can be seen in the learning curves. Typically, ANN training continues as long as the error on the evaluation data decreases over time. If the error stagnates or increases again, it can be assumed that no further abstraction is possible or that the ANN is overfitted. For abstraction and to prevent overfitting, a sufficiently large training data set is necessary. The learning curves of the NEAT algorithm for the postures of the back and arms show typical learning curves on the evaluation data, which stagnate in the course. The training data contain too many contradictions or ambiguities for further abstraction to be possible. For the postures of the legs, the algorithm succeeds only to a very limited extent in formulating abstract regularities of the data in the ANN.

**Fig. 8:**
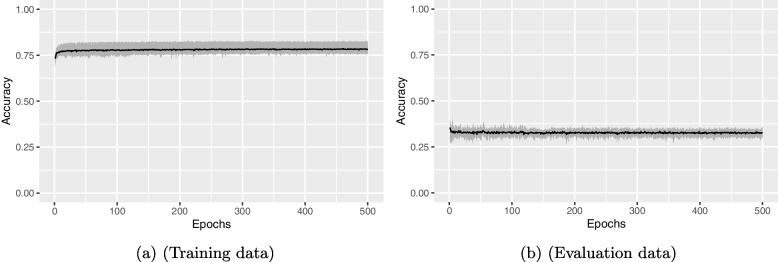
Cumulative results of the ten TensorFlow runs for the arm poses. The black line is the average of the ten runs, the gray area shows the minimum and maximum values of the ten runs

It was to be examined whether the objectively insufficient classification accuracy has its cause in the NEAT learning algorithm used. As a comparison, a conventional MLP network was created using TensorFlow and trained in ten runs and randomly selected training data. The number of neurons as well as their activation functions were chosen based on the networks generated with NEAT. The results in Fig. 8 clearly show that the learning algorithm fails to abstract on the data. The accuracy stagnates at a certain level and starts to decrease slightly at about 250 epochs, which is a sign of network overfitting. However, the accuracy on the training data is also well below the optimal value 1.0 at about 0.75, so one can assume unrecoverable inconsistencies in the data.

#### Final error consideration

In summary, the inconsistent training data are considered to be crucial for the comparatively low classification accuracy of the ANN. The other errors discussed probably have only a minor influence on the result.

Three approaches could be considered for improving the classification accuracy:

1. More training data: if the amount of training data is increased, it can be assumed that the algorithms will succeed in finding additional patterns and combinations in the conflicting data. This improves the error tolerance and possibly the accuracy of the classification.
2. Combining classes: it might be possible to combine the training data for the ML algorithm for hard-to-differentiate individual postures that were detected by human observers only with comparatively low agreement (such as the leg postures xx3 “stand/load on extended leg” and xx5 “stand/load on bent leg”). This could usefully increase the amount of training data and thus improve the overall classification accuracy of the ML model. However, the hard-to-distinguish postures have different OWAS action classes (see Table 5) and thus cannot be meaningfully combined in this case [13].
3. Model constraints on the skeleton: since, strictly speaking, the SIRKA system does not measure body movements but clothing movements, it might be useful to formulate constraints on skeletal movements. For example, movements of the spine (such as a folding of individual vertebrae) appear in the measured data, which are possible for the clothing, but would be fatal for a person if the skeleton were deformed in this way. If one defines the skeleton model with anatomically possible motion radii and maps the motion data to this skeleton, such actually impossible motions of the skeleton would not appear in the training data.

### Appraisal of the NEAT approach

In the previous sections, the diverse possible sources of error have been comprehensively discussed. It remains to discuss the advantages and disadvantages of the evolutionary NEAT approach compared to the usual methods for training.

First, it should be noted that the authors are not aware of any automated deterministic method to construct the structure and topology of a ANN. The usual way is to construct the ANN from layers and units by humans with the specific domain knowledge. Thus, the automated meta-heuristic approach of NEAT has no deterministic counterpart.

When manually constructing a ANN, one chooses the subsequent layers starting from the dimension of the input values. The layers are often arranged in a V-shape, i.e., the layers are arranged in order of size so that as the network is traversed, the dimensionality is gradually reduced. Choosing the number of neurons or combined processing units (e.g., LSTM units) requires experience and manual trial-and-error adjustment (basically, a human meta-heuristic process). If one chooses the network too large, there is a risk of overfitting; if the network is too small, it may not be possible to learn properly (underfitting; possibly the cause of the poor performance of the backpropagation-trained ANN, see above). These problems do not exist in NEAT, where the network is built incrementally. It only grows if it is helpful for adapting the network to the problem to be solved. The prerequisite here is that the training and evaluation data are partitioned in a meaningful way.

The evaluation of large ANN requires considerable computational effort (essentially matrix multiplications). If the ANN is used on mobile systems, the computational effort required is also a factor in the energy consumption of the system. Small ANNs are thus more energy efficient and preferable for this application domain. The networks created with NEAT are as small as possible and as large as necessary. They grow incrementally only as long as is beneficial to the performance of the network.

The advantages of NEAT do not come without drawbacks: because of the parallel optimization of an entire population of ANNs, running the NEAT algorithm to create an optimal ANN is generally more computationally expensive than backpropagation training a single ANN.

## Conclusion

In this paper, an approach has been presented on how intelligent workwear can automatically classify the different postures of the clothing wearer into different categories. Such intelligent workwear could be used in perspective to unobtrusively detect the risk for musculoskeletal disorders of employees based on body postures during work shifts. We demonstrated the complete process of creating a classifying model from recording sensor data, deriving body postures based on an abstract skeletal representation, to training a classifying model.

The SIRKA motion sensing system equipped with 15 inertial sensor nodes has been described as an integral part of such workwear, the data from which will be used for classification. As a special feature, this system is not directly attached to the skin or limbs as is usually the case, but was integrated into the actual work clothing. The resulting inaccuracies were a challenge for the learning algorithm of the classification models. However, this realistic approach was necessary with regard to later use in practice.

Posture data from the abstract skeleton representation were used with the NEAT evolutionary learning algorithm to build artificial neural network-based models for classifying postures using the OWAS method. NEAT generates minimal ANNs, which is beneficial for classification using mobile devices.

The OWAS method for classifying postures, which is usually performed using pen and paper, does not specify fixed angles for the back or knees, for example, so reference postures were needed here as training data for the classification models. For this purpose, reference postures were recorded in a study with human participants, each of which was rated by human observers using OWAS. These ratings served as the ground truth for the learning algorithm. The trained classification models were compared to these ratings to determine their accuracy.

Since the classification results still leave room for optimization, numerous potential sources of error were identified and discussed, which we consider helpful for further approaches in this direction. A detailed ablation analysis as well as an investigation of the more advanced NEAT variants such as HyperNEAT or CoDeepNEAT could help to improve the results and should be investigated next.

## Supplementary Information

**Additional file 1.**

## Data Availability

The datasets generated during and analyzed during the current study are not publicly available due to limitations of ethical approval involving the patient data and anonymity but are available from the corresponding author on reasonable request. A publication of the fully anonymized data in a generally accessible repository is intended.

## Abbreviations

ANN: Artificial Neuronal Network
HPC: High Performance Computing
SIRKA: Sensoranzug zur individuellen Rückmeldung körperlicher Aktivität (Sensor suit for individual feedback of physical activity)
NEAT: Neuroevolution of Augmenting Topologies
OWAS: Ovako Working postures Analysing System
RNG: Random Number Generator

## Footnotes

1 One meta-study showed zero to only small effects [15], although few controlled studies show effects for single body regions or specific training approaches [16, 17].

2 The authors are not aware of any empirical study on the proportion of OWAS among all assessment methods used. However, there is anectdotal evidence as in [21] that assumes widespread use of OWAS.

3 *Unbalanced* in this context means that the classes to be recognized are not uniformly present in the training dataset. Later in this paper, the problem of balancing training data is discussed.

4 For details on sensor fusion without magnetometers, please refer to the work of F. Wenk [41].

5 The matrices are orthogonal with determinant 1 and form the *Special Orthogonal Group of Dimension 3*, see https://mathworld.wolfram.com/SpecialOrthogonalGroup.html.

6 See also https://mo.mathematik.uni-stuttgart.de/kurse/kurs10/seite111.html and http://peheko.netfast.org/m1303/rot3d/drehungen.htm

7 Consider the number of classes of classifiers to be distinguished: $72 = 4 \cdot 3 \cdot 6 > 4 + 3 + 6 = 13$

8 see https://uol.de/fk5/wr/hochleistungsrechnen/hpc-facilities/carl/

9 see https://github.com/chrlns/yahni

10 see https://scikit-learn.org/
