# Supplementary material for: Classification of body postures using smart workwear
Source: BMC Musculoskelet Disord. 2022 Oct 18;23:921. doi: 10.1186/s12891-022-05821-9 (PMC9580122; doi:10.1186/s12891-022-05821-9)
Supplement: Supplementary file 1 — Additional file 1. [file 12891_2022_5821_MOESM1_ESM.pdf]

Appendix

1.1 NEAT Algorithm

Evolutionary algorithms have already been used to optimize the connection weights of ANNs with fixed structure, for example with Differential Evolution [1]. However, the structure of a ANN also affects the performance and trainability of the network [2], even if in principle a fully-connected ANN with only one hidden layer can approximate any continuous function [3]. Algorithmic adaptation of the topology of ANN is also necessary because manual adaptation works only by trial and error and thus involves considerable human effort [4, 2]. The class of algorithms or ANNs in which both topology and weights are evolved is called *Topology and Weight Evolving Artificial Neural Networks* (TWEANNs).

The NEAT algorithm (*Neuroevolution of Augmenting Topologies*) [2], introduced by Stanley and Miikkulainen in 2002, belongs to this class and uses direct coding to represent the nodes and connections of the ANN in the genome. Here, NEAT uses two separate genomes for neurons and connections for genome coding (see Figure 1) [5, p. 17].

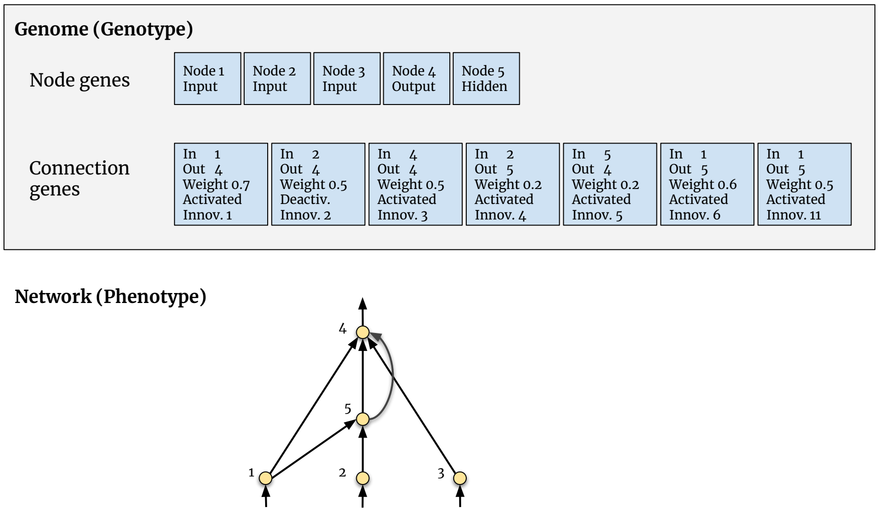

Figure 1: An example of a genotype and its mapping to the ANN as a phenotype (image source: recreated from [2]).

NEAT has three essential characteristics [2]:

- **minimal topology**: The NEAT algorithm initially initializes the population with the smallest possible complexity, i. h., the ANNs have only one input and one output layer at the beginning, whose number of neurons is given by the use case. The algorithm adds new neurons and connections

between neurons in its mutation step. Thus, more complex and different individuals are created with each generation. This principle is called complexification.

- **tracking topology changes**: But if different topologies, including different numbers of connections and neurons, are evolved, how can related gene segments be combined in a one-dimensional vector? The NEAT algorithm solves this problem with a global *Innovation Number* assigned to each gene that is added. The assigned number is also passed on, so that matching genes in the genome for the fusion can still be found in the offspring of subsequent generations. The number of matching genes in a genome also determines the similarity between individuals, which has great significance for the next characteristic.

- **species**: When ANNs mutate in NEAT, i.e., complexity increases due to added neurons and connections, it is likely that the fitness of the ANN will initially decrease before the connection weights are trained to the new topology. To prevent the mutant individuals from being displaced again in the short term by simpler competitors due to their initial weakness, NEAT introduces the concept of *species*. If individuals strongly resemble each other, i. h. they have a very similar genome, then they are combined into one species. Inheritance and selection then take place only within a species, ensuring that topologically different individuals do not displace each other, but only weaker individuals of their own species. With this concept, co-evolution is possible in NEAT, i. h. the parallel optimization of different solution approaches over many generations.

The NEAT algorithm can be written as pseudocode as follows (according to [2] and from YAHNI[1]):

Algorithm 1 NEAT algorithm

```
1: Population ← {NP random individuals, only input and output nodes}
2: while not termination criterion met do
3:     Determine the fitness of each individual in the population
4:     Remove individuals with fitness = 0
5:     Parts population in species
6:     Choose best individuals as elite
7:     Produce descendants
8:     Mutation: add random neuron genes or connection genes
```

In line 3, an evaluable ANN is generated from each individual (genotype) and evaluated against the test data set. If the fitness of the ANN is minimal (fitness = 0), the individual is removed from the population (line 4).

In line 5, the population is divided into different species based on a distance measure and a threshold. At the beginning, the entire population belongs to

[1] See https://github.com/chrlns/yahni.git (YAHNI stands for *Yet Another HyperNEAT Implementation*)

one species and diversifies in the course of the algorithm. The best individuals of a species are protected as elite for the next generation (line 6).

Within each species, offspring are generated in the next step (line 7). The species concept ensures that only compatible individuals are recombined.

At the end of the run (line 8), neuron or connection genes are then randomly inserted into the respective genome. The temporarily generated ANNs are discarded, only the genotype remains over a generation run.

1.2 Balancing Algorithm

The flow of Algorithm 2 is as follows: the main function `BalanceData` receives the datasets S structured by subjects and a minimum tolerated balancing error `balanceVTR`. First, we determine how many records are minimally available from each subject (line 4). Half of this number (`minSamples`) is randomly chosen from each person's records and added to the balanced dataset `BD` (lines 5-8). In this process, the class probabilities from the target sizes of the datasets are summed (variable c). Thus, the datasets are balanced with respect to the subjects*.

However, because of random subsampling, the classes may not be uniformly balanced, i.e., individual classes appear disproportionately often in the dataset. Therefore, one dataset is now randomly selected in turn from each person's dataset. If the data set improves the balance of the classes, it is added to the balanced data set BD, otherwise it is discarded. Depending on the design of the datasets and the class probabilities, this section does not terminate reliably, i.e., it may not be possible to achieve a complete balance. Therefore, `maxIter` ensures that the algorithm terminates eventually.

1.2.1 Choice of hyperparameters for NEAT

The NEAT algorithm can be influenced by a large number of parameters (hyperparameters). Table 1 lists the most important parameters and their values used here.

The most important hyperparameters are the population size, i. i.e. how many neural networks are evolved in parallel, and the number of generations the algorithm runs through. Here, 500 was chosen as the population size. The rule of thumb "ten times the dimension" [6, 7] was interpreted more generously here because of the additional diversity of possible mutations. The maximum number of generations was chosen to be $G_{max} = 2000$, which in preliminary tests proved to be just about feasible with respect to the runtime on the cluster.

Algorithm 2 Balancing the training data

Precondition: abs(x) Absolute value of x
Precondition: len(x) Number of elements in x

```
function BalanceData(S, balanceVTR = 0.01, maxIter = 100000)
    c ← []
    BD ← []                                             ▷ The balanced data
    minSamples ← MinSamples(S) / 2

    for subject in S do                            ▷ Balance the participants
        D ← chose random minSamples from subject
        c ← c + D_out                         ▷ Sum up the class probabilities
        BD ← BD ∪ D

    balance ← Balance(c)
    while balance > balanceVTR and maxIter ¿ 0 do▷ Balance the classes
        maxIter ← maxIter - 1
        for subject in subjects do
            D ← chose random sample from subject
            c_neu ← c + D_out
            if balance > Balance(c_neu) then    ▷ If D contributes to better balance...
                balance ← Balance(c_neu)
                c ← c_neu
                BD ← BD ∪ D
    return BD

function MinSamples(S)        ▷ What is the minimum number of samples each person provides?
    minSamples ← len(S_0)
    for i = 1 to i = N − 1 do
        minSamples ← min(minSamples, len(S_i))
    return minSamples

function Balance(c)
    diff ← 0
    sum ← c_0
    for i = 1 to i = N − 1 do
        diff ← diff + abs(c_i − c_{i−1})
        sum ← sum + c_i
    return diff / sum
```

Table 1: Used hyperparameters for the NEAT algorithm in the YAHNI implementation.

**Parameter**	**Werte**	**Beschreibung**
num.generations (G_{max})	5000	Number of generations that the algorithm runs
popul.size (NP)	1000	Number of different ANN that the algorithm evolves
weight.max	50	Upper limit for initialization of connection weights
weight.min	-50	Lower limit for initialization of connection weights
add.neuron.mutation.rate	0,10	Probability with which a new neuron is added
add.connection.mutation.rate	0,30	Probability with which a new connection is added
training.evalSplit	0,10	Proportion of training data that is reserved for evaluation
recurrent	disallowed	Cycles in the network are not allowed
learningRate	0,01	The learning rate used in the backpropagation method
fullyConnected	true/false	Initial networks with or without complete connections between neurons
num.hidden.neurons	Number	Initial number of hidden neurons

References

[1] Ilonen, J., Kamarainen, J.K., Lampinen, J.: Differential evolution training algorithm for feed-forward neural networks. Neural Processing Letters **17**(1), 93–105 (2003). doi:10.1023/A:1022995128597

[2] Stanley, K.O., Miikkulainen, R.: Evolving neural networks through augmenting topologies. Evolutionary Computation **10**(2), 99–127 (2002). doi:10.1162/106365602320169811

[3] Cybenko, G.: Approximation by Superpositions of a Sigmoidal Function. Approximation Theory and its Applications, 303–314 (1989). doi:10.1007/BF02551274

[4] Gruau, F., Whitley, D., Pyeatt, L.: A comparison between cellular encoding and direct encoding for genetic neural networks. In: Koza, J.R. (ed.) Proceedings of the 1st Annual Conference on Genetic Programming, p. 568 (1996)

[5] Omelianenko, I.: Hands-On Neuroevolution with Python, p. 359. Packt Publishing Ltd., Birmingham (2019)

[6] Chen, S., Montgomery, J., Bolufé-Röhler, A.: Measuring the curse of dimensionality and its effects on particle swarm optimization and differential evolution. Applied Intelligence **42**(3), 514–526 (2015). doi:10.1007/s10489-014-0613-2

[7] Storn, R.M.: On the usage of differential evolution for function optimization. Proceedings of North American Fuzzy Information Processing, 519–523 (1996). doi:10.1109/NAFIPS.1996.534789
